# Association of Copy Number Variation at Intron 3 of *HMGA2* With Navel Length in *Bos indicus*

**DOI:** 10.3389/fgene.2018.00627

**Published:** 2018-12-07

**Authors:** Tamíris Sayuri Aguiar, Rafaela Beatriz Pintor Torrecilha, Marco Milanesi, Adam Taiti Harth Utsunomiya, Beatriz Batista Trigo, Abdulfatai Tijjani, Hassan Hussein Musa, Flávia Lombardi Lopes, Paolo Ajmone-Marsan, Roberto Carvalheiro, Haroldo Henrique de Rezende Neves, Adriana Santana do Carmo, Olivier Hanotte, Tad Stewart Sonstegard, José Fernando Garcia, Yuri Tani Utsunomiya

**Affiliations:** ^1^Department of Preventive Veterinary Medicine and Animal Reproduction, School of Agricultural and Veterinarian Sciences, São Paulo State University, Jaboticabal, Brazil; ^2^Collaborating Centre on Animal Genomics and Bioinformatics, International Atomic Energy Agency, Araçatuba, Brazil; ^3^Department of Support, Production and Animal Health, School of Veterinary Medicine, São Paulo State University, Araçatuba, Brazil; ^4^Department of Animal Science Food and Nutrition and Biodiversity and Ancient DNA Research Center – BioDNA, Università Cattolica del Sacro Cuore, Piacenza, Italy; ^5^Cells, Organisms and Molecular Genetics, School of Life Sciences, University of Nottingham, Nottingham, United Kingdom; ^6^Faculty of Medical Laboratory Sciences, University of Khartoum, Khartoum, Sudan; ^7^Department of Animal Science, School of Agricultural and Veterinarian Sciences, São Paulo State University, Jaboticabal, Brazil; ^8^GenSys Consultores Associados, Porto Alegre, Brazil; ^9^Escola de Veterinária e Zootecnia, Universidade Federal de Goiás, Goiânia, Brazil; ^10^LiveGene – CTLGH, International Livestock Research Institute, Addis Ababa, Ethiopia; ^11^Recombinetics, Inc., Saint Paul, MN, United States

**Keywords:** bovine, SNP, haplotype, CNV, *HMGA2*, *PLAG1*, *IGF2*

## Abstract

Navel injuries caused by friction against the pasture can promote infection, reproductive problems and costly treatments in beef cattle raised in extensive systems. A haplotype-based genome-wide association study (GWAS) was performed for visual scores of navel length at yearling in Nellore cattle (*Bos indicus*) using data from 2,016 animals and 503,088 single nucleotide polymorphism (SNP) markers. The strongest signal (*p* = 1.01 × 10^-9^) was found on chromosome 5 spanning positions 47.9–48.2 Mbp. This region contains introns 3 and 4 and exons 4 and 5 of the high mobility group AT-hook 2 gene (*HMGA2*). Further inspection of the region with whole genome sequence data of 21 Nellore bulls revealed correlations between counts of the significant haplotype and copy number gains of a ∼6.2 kbp segment of intron 3 of *HMGA2*. Analysis of genome sequences from five African *B. indicus* and four European *Bos taurus* breeds revealed that the copy number variant (CNV) is indicine-specific. This intronic CNV was then validated through quantitative polymerase chain reaction (qPCR) using Angus animals as copy neutral controls. Importantly, the CNV was not detectable by means of conventional SNP-based GWAS or SNP probe intensity analyses. Given that *HMGA2* affects the expression of the insulin-like growth factor 2 gene (*IGF2*) together with the pleomorphic adenoma gene 1 (*PLAG1*), and that the latter has been repeatedly shown to be associated with quantitative traits of economic importance in cattle, these findings highlight the emerging role of variants impacting the insulin-like growth factor pathway to cattle breeding.

## Introduction

Navel length at yearling (NY) is an economically important trait in beef cattle raised in extensive production systems. A pendulous navel increases the risk of injuries caused by friction against the pasture, leading to infection, reproductive impairment and treatment expenses (Ashdown, 2006; Rabelo et al., 2008; Boligon et al., 2016). This trait is especially important to cattle raised in Brazil, the second largest beef exporter of the world, where Nellore (*Bos indicus*) animals compose the majority of the herds and approximately 88% of the females in reproductive age are subjected to natural mating (Asbia, 2016). Consequently, visual scores of navel length are routinely recorded in national commercial breeding programs together with other growth traits such as body weight and carcass visual scores (Neves et al., 2014).

A recent genome-wide association study (GWAS) for body weight and carcass visual scores in Nellore cattle revealed pleiotropic loci sheltering genes that are known to modulate the insulin-like growth factor (IGF) or somatomedin pathway (Pereira et al., 2016). In particular, the locus containing the pleomorphic adenoma gene 1 (*PLAG1*), which is a transcription factor for the insulin-like growth factor gene 2 (*IGF2*), contributed significantly to the genetic variance of all traits analyzed. Indeed, the *PLAG1* chromosomal domain has been shown to affect a wide range of economically relevant traits in both *Bos taurus* (Karim et al., 2011; Bolormaa et al., 2014; Saatchi et al., 2014) and *B. indicus* (Fortes et al., 2013; Utsunomiya et al., 2013; Pereira et al., 2016) cattle. However, genome-wide scans for visual scores of navel length were not yet reported in *B. indicus* cattle, leaving opened the question of whether polymorphisms in *PLAG1* or any other modulator of the IGF pathway also contribute to the genetic variance of navel length in that species.

Here we report a haplotype-based GWAS for visual scores of navel length at yearling (NY) in Nellore cattle, which resulted in the discovery of a copy number variation (CNV) at intron 3 of *HMGA2*, another transcription factor for *IGF2* that was not detectable from conventional GWAS or SNP probe intensity analyses. Moreover, validation of the CNV through quantitative polymerase chain reaction (qPCR) showed that the genome-wide significant haplotype performed well as a predictor of CNV genotype in our samples. Finally, analysis of a panel of whole genome sequences of African *B. indicus* and European *B. taurus* breeds revealed the CNV to be of *B. indicus* origin.

## Materials and Methods

### Genotypes

Illumina BovineHD BeadChip assay (786,799 SNPs) genotypes of 953 Nellore bulls and 1,278 Nellore cows were available for analysis from previous studies (Carvalheiro et al., 2014; Zavarez et al., 2015). These animals were born between 1963 and 2008, parented over ten Nellore generations, and comprised part of the genomic selection reference population from a commercial breeding program that routinely performs genetic evaluations for weight, carcass and reproductive traits. All genotyped samples had a minimum genotyping rate of 90%. Autosomal markers with unique genomic coordinates were filtered with PLINK v1.90b4.6 (Purcell et al., 2007; Chang et al., 2015) for a minimum call rate of 95%, GenTrain Score of at least 70% and minor allele frequency of at least 2%. The filtered genotypes resulted in a reduced set of 503,088 markers, which were retained for phasing with the Segmented HAPlotype Estimation and Imputation Tool (SHAPEIT2) v2.r837 (O’Connell et al., 2014). Phasing was performed with a burn in of 10 iterations, pruning of 10 iterations, 50 main iterations, 200 states, windows of 500 kbp and effective population size of 113. The latter parameter was estimated from genotype data with SNeP v1.1 (Barbato et al., 2015).

### Phenotypes

Pseudo-phenotypes were based on estimated breeding values (EBVs) obtained from an animal model fitted to records of 745,466 animals. Navel length at yearling (NY) was recorded based on visual evaluation of animals by trained technicians. The navel length scores were assigned considering an absolute scale, ranging from 1 to 5, so that the largest scores were attributed to animals with longer or more pendulous navels. For males, the penile sheath was considered part of the navel. Animals pertaining to the same contemporary group (i.e., animals from the same herd, sex, birth year, birth season and management group) were evaluated by the same technicians, so that the fixed effect of contemporary group fitted in the animal model was expected to account for any systematic effects associated to technicians. The distribution of NY phenotypes is presented in the Supplementary Figure S1. Prior to GWAS, EBVs were deregressed following Garrick et al. (2009) and filtered for a minimum accuracy of 0.70. This filter was used to reduce influence of heterogeneity of variance in the GWAS analysis and to ensure that the pseudo-phenotype of each animal was mainly based on own or progeny phenotypes, rather than information from parents or siblings. Therefore, the GWAS analysis was based on a subset of 2,016 animals.

### Genome-Wide Association Analysis

The GWAS model used here was essentially the leave-one-chromosome-out (LOCO) procedure proposed by Yang et al. (2014), except that haplotypes were tested instead of single SNPs. Model fitting was performed in GCTA v.1.90.2 beta (Yang et al., 2011) by using haplotype data as input via coding of haplotypes as pseudo-SNP markers. Theoretical support to this approach was previously detailed by Da (2015). More specifically, haplotype data were provided to GCTA in binary (bed/bim/fam) format with genotypes expressed in terms of number of copies of each haplotype (0, 1, or 2). The association analysis for each haplotype was then based on the following mixed linear model:

$$y\;=\;1_{n}\mu +xb+u+e$$

where y is the vector of deregressed EBVs, 1_n_ is a vector of ones (length equal to *n*, the number of animals with deregressed EBV), μ is the intercept, × is a *n* × 1 vector of standardized haplotype counts, ***b*** is the fixed effect of the haplotype, u is the *n* × 1 vector of accumulated contributions of random effects of all SNPs except those located in the same chromosome as the haplotype being tested (i.e., polygenic scores), and e is the *n* × 1 vector of random residual effects. It was assumed that y ∼ MVN (**1**_**n**_μ + **x***b*, **G**σ_**u**_^2^ + **I**σ_**e**_^2^), where **G** is the SNP-based genomic relationship matrix (GRM) excluding all SNPs pertaining to the same chromosome where the tested haplotype is located, σ_**u**_^2^ is the marked additive genetic variance, **I** is an identity matrix and σ_**e**_^2^ is the residual variance. The model above was fitted in two steps: first, variance components were estimated using the average information restricted maximum likelihood (AI-REML) algorithm; then, estimates of fixed effects were obtained using generalized least squares equations. In order to reduce computational burden, variance components were estimated only once per chromosome using a reduced model without haplotypes. By conditioning all analyses on the accumulated effects of SNPs, associations presumably reflected significant haplotype effects not captured by random SNP effects. Moreover, the SNP-based GRM helped controlling the analyses for putative confounding effects of cryptic relationships and population substructure. Haplotypes were constructed with GHap v1.2.2 (Utsunomiya et al., 2016) using overlapping segments of six consecutive markers (i.e., sliding windows of six markers moving one marker at a time), considering that the average intermarker distance was ∼5 kbp and that linkage disequilibrium extends up to 30 kbp in the Nellore genome (Espigolan et al., 2013). Only haplotypes with frequency between 5 and 95% were tested (analogous to exclusion of loci with minor allele frequency below 5% in conventional GWAS). Additionally, to assess the sensitivity of the GWAS procedure to the choice of haplotype size, we repeated the analysis with haplotypes of 1 (equivalent to single marker analysis), 5 and 10 SNPs. Haplotypes were prioritized for investigation if their *p*-values were lower than a stringent Bonferroni corrected significance level of 0.05/*N*, where *N* is the total number of tested haplotypes.

### Association With Additional Traits

The most significant haplotype in the GWAS analysis was further subjected to a screening for pleiotropic effects by taking advantage of deregressed EBVs that were also available for nine additional traits, namely: (1) birth weight, (2) weight gain from birth to weaning, (3) weight gain from weaning to yearling, (3) scrotal circumference, (4) age at first calving, (5) gestation length, and visual scores for (6) conformation, (7) precocity, and (8) muscling at yearling. These traits are routinely recorded and used as part of the genetic evaluation system of the commercial breeding program, and the models used to obtain deregressed EBVs for these traits were previously described by Neves et al. (2014) and Pereira et al. (2016). For each trait, pseudo-phenotypes were regressed onto haplotype counts using linear regression, and effects were considered significant if the nominal *p*-value was smaller than 0.05. Following the same rationale as in the GWAS analysis, only animals presenting accuracy of deregressed EBV greater than 0.70 were analyzed. In order to evaluate potential interference in the regression analysis that could be caused by unaccounted heterogeneity of residual variance, we paralleled all analyses with weighted linear models using the weights proposed by Garrick et al. (2009).

### Analysis of Whole Genome Sequence Data

Next-generation sequence data of Nellore animals were obtained from a previous study (Utsunomiya et al., 2017) and consisted of Illumina HiSeq 2000 paired-end reads from 21 bulls aligned to the UMD v3.1.1 *B. taurus* assembly (Zimin et al., 2009). The average sequencing coverage was 9.25×. For the candidate region detected by the GWAS analysis, single nucleotide variants and short insertions/deletions were identified with the mpileup algorithm from SAMtools v1.3.1 (Li et al., 2009) and annotated with the Ensembl Variant Effect Predictor (VEP) (McLaren et al., 2016). Copy number gains were identified by increase of nucleotide coverage as compared to the average sample coverage. First, for each sample and nucleotide, coverage was computed as *N*_reads_/C, where *N*_reads_ is the number of reads encompassing the nucleotide and *C* is the sample coverage. Then, bulls were grouped according to their number of copies (0, 1, and 2) of the significant haplotype and nucleotide coverage was averaged within groups. A last normalization step was adopted by taking the median coverage of 1 kbp windows. This procedure was intended to smooth out outlying values and increase the signal-to-noise ratio in the data. Candidate variants and alignment data were also inspected with the Integrative Genomics View (IGV) v2.3 software (Robinson et al., 2011; Thorvaldsdóttir et al., 2013). Occurrence of the candidate variant in other cattle breeds was investigated using re-sequencing data (*n* = 13) from five African zebu *B. indicus* cattle breeds, namely Kenana, Butana, Sudanese Baggara, Ogaden, and Kenyan Boran (Kim et al., 2017), as well as publicly available whole genome sequence data (*n* = 20) of four *B. taurus* breeds (Angus, Holstein, Jersey, and Simmental) (Daetwyler et al., 2014). Per sample sequencing depth and number of animals per breed are provided in the Supplementary File S1. Comparative genomics and orthology analyses were performed using the UCSC (Casper et al., 2018) and Ensembl (Zerbino et al., 2018) genome browsers.

### SNP Probe Intensity Analysis

Let X and Y be the normalized signal intensity for the A and B allele, respectively, and *R* = X + Y. The normalized total signal intensity (LRR) was calculated as log_2_ (R_observed_/R_expected_), where R_expected_ was computed from linear interpolation of the canonical genotype clusters. Since LRR is prone to genomic “waves,” supposedly produced by differences in GC-content (%GC) and intensified as the sample deviates from optimal DNA quantity (Diskin et al., 2008), LRR values were further corrected for waviness to avoid false positive copy number variation. Adjustment was achieved by regressing LRR values onto %GC computed over 1 Mbp windows encompassing each marker and taking residuals as the new waviness-normalized LRR values. Calculation of %GC from a FASTA file of the UMD v3.1.1 assembly was performed with the Nuc program in BEDTools v2.26.0 (Quinlan, 2014). Values of LRR were then averaged across haplotype classes 0, 1, and 2 and compared with coverage data obtained from whole genome sequences.

### qPCR

A qPCR assay was set up in order to validate a CNV that was identified in the whole genome sequence analysis. The pair of primers 5′ TCAAAGCCAACTGATTGCTG 3′ (forward) and 5′ TTTCCTATGGTCCCAAGCG 3′ (reverse) was designed considering the limits of the CNV region using the NCBI Primer-BLAST^1^. Primers 5′ GGTGCCTTGTGGGAGATGTA 3′ (forward) and 5′ GTGTCAGCTGCCTGAGTCCT 3′ (reverse) targeting the tumor suppressing subtransferable candidate 4 gene (*TSSC4*) were used as a single copy reference. The assay was performed using a 96-samples plate. The qPCR analysis was then conducted using samples available from our DNA bank, and comprised 4, 4 and 3 animals with 0, 1, and 2 copies of the significant haplotype, respectively. Additionally, 4 Angus animals were used as copy neutral controls. All samples were assessed in triplicate and no-DNA templates were included for each pair of primers as a control. Each assay consisted of a reaction with a total volume of 20 μl, which contained 1 μl of genomic DNA, 0.8 μl of each primer (10 μM), 7.4 μl of pure water and 10 μl of QuantiTect^®^ SYBER^®^ Green RT-PCR Master Mix (QIAGEN^®^). Relative increase of copy number was calculated for each sample using the 2^-ΔΔCt^ method (Livak and Schmittgen, 2001). The 2^-ΔΔCt^ calculation considered as the calibrator sample one Angus animal.

## Results

### GWAS Identifies a *HMGA2* Haplotype Associated With Navel Length at Yearling in Nellore Cattle

**FIGURE 1:**
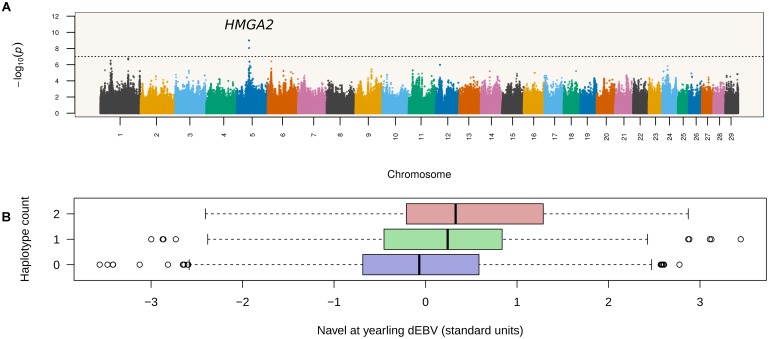
Haplotype-based GWAS maps navel length at yearling associations to a locus containing *HMGA2*. **(A)** Each point in the Manhattan plot corresponds to a six-marker long haplotype. The dashed horizontal line corresponds to the Bonferroni threshold (*p* < 2.44 × 10^-8^). **(B)** Distributions of deregressed EBVs according to number of copies of the leading haplotype. The *HMGA2* locus accounted for 2.04% of the variance in deregressed EBVs.

A total of 2,048,168 six-marker haplotypes were tested for association with NY in Nellore cattle. The minimum, median and maximum number of observed haplotypes per six-marker window was 1, 4, and 15, respectively. Association results pointed to a single leading QTL region on chromosome 5 mapping between 47.9 and 48.2 Mbp (Figure 1). Four haplotypes from consecutive segments spanning positions 47,921,750–48,069,099 bp were tied as the most significant haplotype (*p* = 1.01 × 10^-9^), which formed the consensus allele TCCTCCAAC. This consensus haplotype overlapped introns 3 and 4 and exons 4 and 5 of *HMGA2*.

### Additional Effects Associated With the *HMGA2* Haplotype

By using a panel of nine additional traits that are routinely recorded by the Nellore commercial breeding program, we investigated whether the *HMGA2* haplotype could also be associated with other economically important traits in Nellore cattle. After fitting regression models for all nine traits, we found weak but significant (*p* < 0.05) associations for visual scores of precocity and muscling at yearling (Table 1). The use of weights in the regression analyses had little impact on the results. Of note, the direction of the effects were opposite to that observed for navel length at yearling, where the TCCTCCAAC haplotype was associated with higher scores for navel length and lower scores for precocity and muscling.

**Table 1:** Effects of the *HMGA2*-CNVR tag haplotype on deregressed estimated breeding values of nine traits in Nellore cattle.

Trait	Sample size	Unweighted regression^a^			Weighted regression^b^		
		Coefficient	*SE*	*p*-value	Coefficient	*SE*	*p*-value
Birth weight	1,993	0.037	0.055	0.505	0.047	0.052	0.362
Weight gain from birth to weaning	2,108	0.325	0.262	0.215	0.309	0.264	0.241
Weight gain from weaning to yearling	2,052	-0.243	0.355	0.494	-0.221	0.356	0.536
Visual score for conformation	2,023	0.030	0.019	0.116	0.026	0.019	0.166
**Visual score for precocity**	**2,022**	**-0**.**045**	**0**.**026**	**0**.**084**	**-0**.**055**	**0**.**026**	**0**.**036**
**Visual score for muscling**	**2,020**	**-0**.**060**	**0**.**025**	**0**.**017**	**-0**.**070**	**0**.**025**	**0**.**005**
Scrotal circumference	1,627	-0.041	0.058	0.474	-0.050	0.058	0.396
Age at first calving	636	0.417	0.526	0.429	0.455	0.445	0.307
Gestation length	1,951	0.077	0.133	0.561	0.145	0.130	0.263


*Significant effects (p < 0.05) are marked in bold.*

*^a^Simple linear regression analysis; ^b^Weighted regression using weights proposed by Garrick et al. (2009).*

### Nellore Sequence Data Reveals an Intronic CNV Correlated With the *HMGA2* Haplotype

**FIGURE 2:**
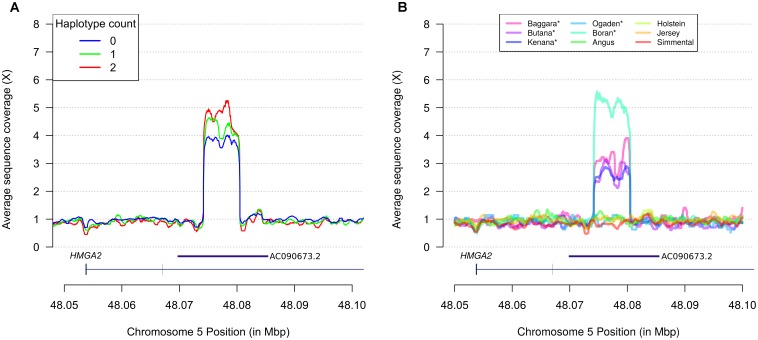
Discovery of a *B. indicus* specific CNV on *HMGA2* affecting navel length at yearling. **(A)** Relative fold increase in sequence coverage of a segment of *HMGA2* intron 3 correlates with TCCTCCAAC haplotype counts in Nellore cattle. Each smoothed curve corresponds to sequence coverage averaged across samples with same haplotype count. **(B)** Inspection of the CNVR in additional European *B. taurus* and African *B*. *indicus* (marked with ^∗^) breeds reveals specificity of copy gains in *B*. *indicus*. The AC090673.2 gene annotation was manually curated and predicted from expressed sequence tags (ESTs) derived from human tissues.

Assuming that the TCCTCCAAC haplotype tagged an unobserved causal variant, we used haplotype counts to predict causal variant genotypes in a sample of 21 Nellore bulls with whole genome sequence data. From a total of 1,185 sequence variants detected by SAMtools within the chromosome 5 range 47.9–48.2 Mbp, only 6 presented genotype correspondence with haplotype counts, namely: rs209832737 (CHR5:47,962,011, C > G), rs521422509 (CHR5:47,964,144, A > G), rs525140201 (CHR5:47,965,249, G > A), rs521794670 (CHR5:47,966,055, G > A), rs519732918 (CHR5:47,966,375, T > C), and rs516281664 (CHR5:47,967,920, C > T). While we could not exclude causality of any of these variants, none of them were novel or intragenic, making it difficult to select any specific variant for further scrutiny. However, analysis of coverage data revealed an additional variant comprising a ∼6.2 kbp CNV spanning positions 48,074,233–48,080,443 (Figure 2A). This CNV was located at intron 3 of *HMGA2*, and TCCTCCAAC haplotype counts 0, 1, and 2 translated into approximate fold changes of 4.0, 4.6, and 5.2 in sequence coverage across the ∼6.2 kbp segment in the Nellore samples. Importantly, all sequenced animals presented copy gains at the CNV region (hereafter denoted *HMGA2*-CNVR), regardless of haplotype count, suggesting a baseline copy gain instead of a copy neutral sequence background, and co-segregation of the relevant haplotype with additional copies of the ∼6.2 kbp DNA segment. Inspection of alignments of the bovine *HMGA2*-CNVR sequence against the human reference genome assembly (GRCh38.p12) showed that the region spanned a *HMGA2* antisense RNA (accession numbers AC090673.2 and ENSG00000256083). The UCSC LiftOver tool further predicted that the antisense RNA would map to positions 48,070,427–48,085,449, which substantially overlapped with the detected CNV. The existence of AC090673.2 in the human genome was well supported by evidence from expressed sequence tags (ESTs) obtained from pooled human melanocyte, fetal heart and pregnant uterus samples (accession number AA773790.1). Therefore, the *HMGA2*-CNVR stood as the most plausible candidate variant underlying the NY associations.

### Genome Sequences From Nine Cattle Breeds Indicate a *B. indicus* Origin for the *HMGA2*-CNVR

In order to determine whether the *HMGA2*-CNVR was a recent derived mutation private to Nellore cattle or an ancient variant, we inspected the candidate chromosomal region in sequence data from five African *B. indicus* and four European *B. taurus* breeds (Figure 2B). Four out of five *B. indicus* breeds presented increased coverage compatible with the *HMGA2*-CNVR previously found in Nellore cattle, whereas all *B. taurus* breeds were copy neutral at the relevant positions on chromosome 5. These results strongly point to an indicine-specific CNV.

### Read Pair Orientation Analysis Solves the *HMGA2*-CNVR Structural Arrangement

**FIGURE 3:**
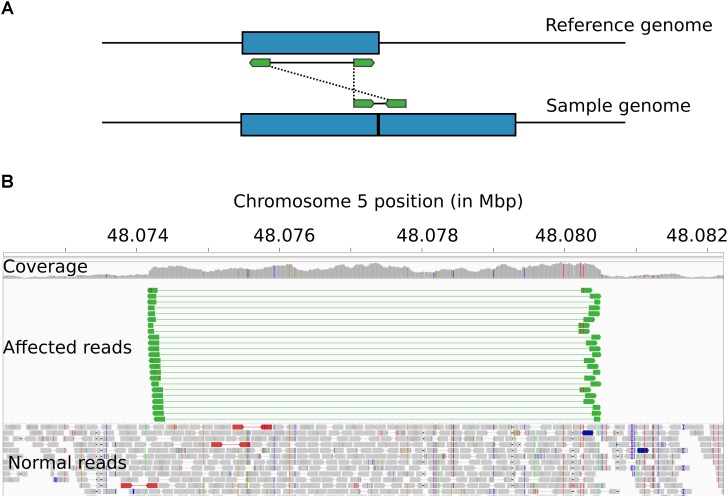
Structural arrangement of the *HMGA2*-CNVR. **(A)** Schematic of the alignment of paired-end reads (in green) derived from an insert encompassing the boundaries of two repeats of a duplicated genomic segment (in blue). When the reads are aligned against the reference genome, the insert appears to be much larger than expected, usually spanning the entire length of the duplicated segment. Additionally, the tandem arrangement causes read pairs to be oriented toward the outer sides of the predicted insert. **(B)** IGV visualization of the *HMGA2*-CNVR in a whole-genome sequenced *B. indicus* animal showing several paired-end reads (in green) satisfying the pattern described above.

Visualization of read alignments in *B. indicus* samples with the IGV software showed inserts with larger than expected sizes that spanned the whole extension of the *HMGA2*-CNVR segment, with paired-end reads oriented to the outer sides of the inserts. This pattern of read pair orientation is indicative of duplications arranged in tandem with structurally identical repeats (Figure 3). Therefore, the *HMGA2*-CNVR structure is most likely explained by a tandem duplication.

### qPCR Validates Haplotype-Based Predictions of CNV Genotypes

The TCCTCCAAC haplotype tag suggested that the frequency of additional copy gains at the *HMGA2*-CNVR in our Nellore sample was 16.5%. The numbers of animals carrying 0, 1, and 2 copies of the tag haplotype were 1,554, 619, and 58, respectively. Based on available samples in our DNA bank, we randomly chose 4 animals from each group for validation of the *HMGA2*-CNVR with qPCR. An exception was the group of TCCTCCAAC-homozygote animals, which included only three samples. We also used DNA from 4 Angus animals as copy neutral controls, assuming that *B*. *taurus* animals were copy neutral as observed in the sequence coverage analysis. The average 2^-ΔΔCt^ values for carriers of 0, 1, and 2 copies of the tag haplotype in the Nellore breed were, respectively, 7.04 ± 1.57, 8.79 ± 1.93, and 9.02 ± 2.79, whereas Angus animals presented an average of 1.39 ± 0.62. The qPCR results were therefore in agreement with the existence of a CNVR located in intron 3 of *HMGA2* in Nellore cattle, for which relative copy gain could be predicted by the TCCTCCAAC haplotype.

### Conventional GWAS and SNP Probe Intensity Data Failed to Detect the *HMGA2*-CNVR

**FIGURE 4:**
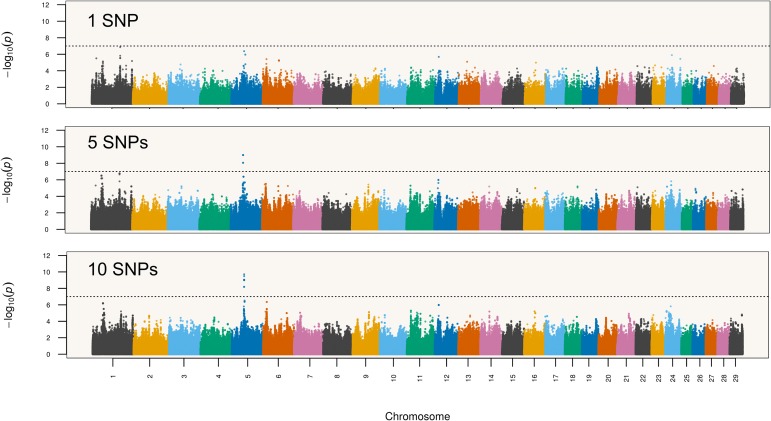
Navel length at yearling associations with varying haplotype sizes (1, 5, and 10 SNPs).

**FIGURE 5:**
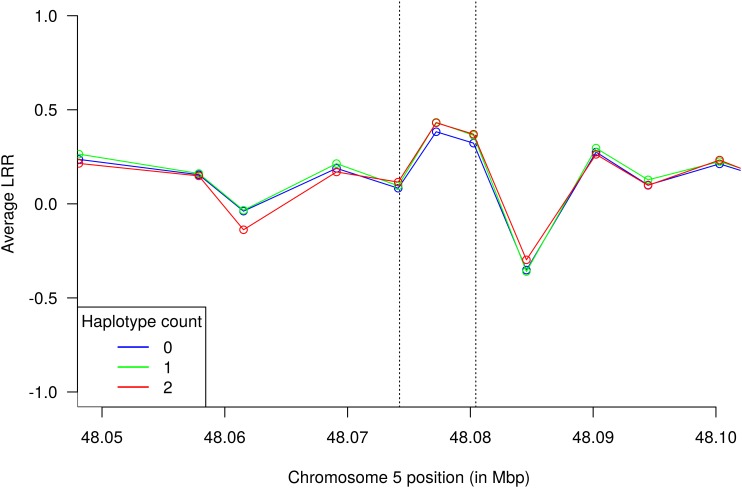
Average probe intensity data from the BovineHD assay for the *HMGA2*-CNVR segment (vertical dashed lines). Each curve corresponds to average LRR values of samples with the same TCCTCCAAC haplotype count. Points in the curves indicate positions of SNP markers.

In order to evaluate sensitivity of GWAS results to the choice of haplotype size, we repeated the genome-wide scan analysis with single SNPs and haplotypes recovered from segments of 5 (1,902,119 tested haplotypes) and 10 markers (2,441,374 tested haplotypes) (Figure 4). Although the topology of the chromosome 5 QTL was fairly preserved across all analyses, the single SNP scan did not present enough power to declare the *HMGA2* region as significant considering a Bonferroni-corrected significance level. This suggests that haplotypes performed better than single SNPs as tags for the putative causal variant in this particular study. Therefore, a conventional single-marker GWAS analysis using Bonferroni correction would have missed the *HMGA2* association reported here. Indeed, associations were even stronger when the haplotype size was increased to 10 markers. Regarding detection of the *HMGA2*-CNVR via probe intensity data, a previous study using the same Nellore genotypes (Zhou et al., 2016) did not detect this CNV with either the CNAM optimal segmentation method from the Golden Helix SVS v8.3.0 software (Golden Helix, Inc., Bozeman, MT, United States) or the Hidden Markov Model (HMM) implemented in PennCNV (Wang et al., 2007). Therefore, we decided to directly inspect LRR values from SNP probes to evaluate whether intensity data could capture information from the CNV. As shown in Figure 5, only two SNP markers mapped to *HMGA2*-CNVR, indicating that the local SNP density in the BovineHD assay was insufficient to reveal the presence of the CNV with confidence. Moreover, only a slight increase in LRR values could be observed for these two SNPs as compared to their neighboring markers, which was probably not high enough to be detectable with either SVS or PennCNV.

## Discussion

The IGF or somatomedin pathway is a complex system that controls the signaling cascades of the PI3K/Akt, MAPK, and Ras/Raf/MEK/ERK pathways, resulting in cell growth and inhibition of apoptosis (Rosenzweig and Atreya, 2010; King and Wong, 2012). The main components of the IGF system, namely *IGF1* and *IGF2*, are preferentially expressed after and before birth, respectively (Bergman et al., 2013). In particular, *IGF2* is maternally imprinted, and disrupting mutations inherited from the male germline cause growth deficiency in mice (DeChiara et al., 1991). Moreover, a mouse knockout model of the pleomorphic adenoma gene 1 (*PLAG1*), a transcription factor for *IGF2*, also presented marked growth retardation and decreased fertility (Hensen et al., 2004). Indeed, a naturally occurring *PLAG1* mutation has been shown to affect a wide range of economically relevant traits in both *B. taurus* (Karim et al., 2011; Bolormaa et al., 2014; Saatchi et al., 2014) and *B. indicus* (Fortes et al., 2013; Utsunomiya et al., 2013; Pereira et al., 2016) cattle, implicating a major contribution of variants impacting the IGF system to genetic variance of several traits in the bovine species.

**FIGURE 6:**
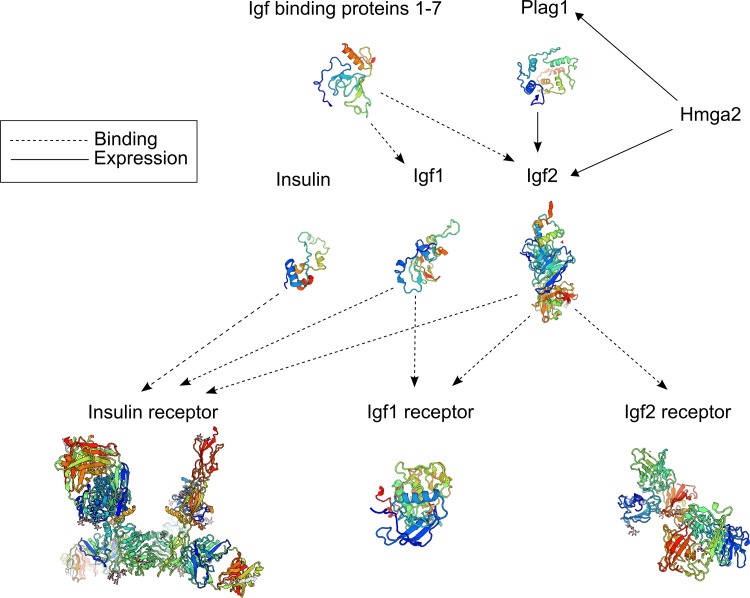
Schematic of the main genes involved in the insulin-like growth pathway. Protein tertiary structures displayed in this figure were built with SWISS-MODEL (Bienert et al., 2017).

The discovery of multiple quantitative trait loci (QTL) mapping to the bovine *PLAG1* chromosomal domain opens the question of whether other regulatory genes and variants affecting the expression of *IGF1* and *IGF2* might also lead to phenotypic differences of economical relevance in cattle. For instance, the major transcription factor affecting the expression of *IGF2* is encoded by the high mobility AT-hook 2 gene (*HMGA2*). Regulation of *IGF2* by *HMGA2* has been recently demonstrated to occur directly or through increased expression of *PLAG1* (Klemke et al., 2014; Abi Habib et al., 2018) (Figure 6). Here, we reported the identification of a CNV located at intron 3 of *HMGA2* that was associated with navel length at yearling (NY) in Nellore (*B. indicus*) cattle. This CNV was detected through a combination of haplotype-based GWAS and whole genome sequence analysis. We also showed that this candidate variant was difficult to identify via conventional GWAS and state-of-the-art CNV detection methods. Moreover, we found that other *B. indicus* breeds apart from Nellore cattle also carry copy gains of this *HMGA2* intronic segment.

Interactions among *HMGA2*, *PLAG1*, and *IGF2*, and their numerous pleiotropic effects on traits related to growth and reproduction, have only recently started to emerge (Klemke et al., 2014; Abi Habib et al., 2018). However, the importance of *HMGA2* as an oncogene, and its regulation by a series of micro RNAs (miRNA) binding to its 3′-UTR region, has been known for at least a decade. For instance, one of the first characterized miRNAs, let-7, is a major negative regulator of *HMGA2* and explains the suppression of this gene in later stages of development (Lee and Dutta, 2007). Another relevant regulatory miRNA is miR-763, which is encoded by intron 3 of *HMGA2* and possibly co-expressed with its host gene (Artzi et al., 2008; von Ahsen et al., 2008). Intron 3 of *HMGA2* is also a frequent target of structural and chromosomal abnormalities in human tumors. The *HMGA2*-CNVR identified in the present study occurs on the same intron. The orthologous sequence in humans is predicted to generate an antisense, long non-coding RNA (AC090673.2), which is yet to be validated and observed in the bovine species. Nevertheless, given the major regulatory role of this intron on *HMGA2* transcript abundance, we propose an effect of variation of copy number at this CNVR on *HMGA2* expression and navel size in *B. indicus* cattle. This is also supported by associations between penile sheath and SNPs close to *HMGA2* in Brahman (*B. indicus*) and Tropical Composite (*B. indicus* ×*B. taurus*) cattle (Porto-Neto et al., 2014), indicating that the *HMGA2*-CNVR might also segregate in these populations.

Apart from the observed effects of the *HMGA2*-CNVR on navel size, the candidate variant is potentially involved with other heritable traits considering the pleiotropic nature of *HMGA2*. For instance, *HMGA2* variants have been associated with floppy ears in dogs (Boyko et al., 2010), ear size in pigs (Li et al., 2012), fat deposition in cattle (Bolormaa et al., 2014) and stature in humans (Weedon et al., 2007; Yang et al., 2010), cattle (Bouwman et al., 2018), horses (Frischknecht et al., 2015), and dogs (Hayward et al., 2016). Mice homozygous for a null *HMGA2* allele present a “superpygmy” phenotype, reduced amounts of fat tissue and infertility (Federico et al., 2014). In the present study alone, we found that other productive traits such as visual scores for precocity and muscling at yearling may also be affected by the candidate variant. Also, the detection of the *HMGA2*-CNVR in African *B. indicus* cattle suggests that the variant might be very old. The *B. indicus* population in Africa descended from importation of animals from Asia during the Arab invasions between the 7th–8th centuries (Hanotte et al., 2002), whereas the Brazilian Nellore population derived from imports of Indian animals in the mid 20th century (Ajmone-Marsan et al., 2010). Altogether, these observations suggest that the candidate variant is probably a wide spread ancient *B. indicus* mutation, which might have had an adaptive value in this subspecies. Given that the hump, the dewlap (i.e., saggy chest and neck skin) and the pendulous navel are phenotypic marks that morphologically differentiate *B. indicus* from *B. taurus* cattle, future analyses of the *HMGA2*-CNVR should address the question of whether this variant had a role in the evolutionary history of the *B. indicus* lineage.

## Data Availability

Whole genome sequences of African and European cattle are available through the NCBI SRA Archive under BioProjects PRJNA312138 and PRJNA238491, respectively. The Nellore datasets are not publicly available because they comprise part of the genomic selection reference population from a commercial breeding program ran by an alliance of cattle breeders from Brazil. Data are, however, available for academic use from the authors upon request (requires a signed Material Transfer Agreement for exclusive research purpose).

## Author Contributions

YU and JG conceived and designed the study. AdC and TS coordinated genotyping and sequencing of Nellore animals. AT, HM, and OH provided and analyzed African cattle sequence data. RC and HN analyzed phenotypes and pedigree data and generated deregressed EBVs. AU performed genotype phasing. TA and YU performed association analyses. MM, YU, and PA-M designed the qPCR experiments. TA, BT, RT, and FL performed the qPCR assays. TA, RT, and MM performed Nellore and *B. taurus* sequence data analyses. TA and YU wrote the manuscript. All authors revised and agreed with the contents of the manuscript.

## Conflict of Interest Statement

HN is employed by Gensys Consultores Associados and TS is employed by Recombinetics, Inc. TS and YU are Associate Editors at Frontiers in Genetics, Livestock Genomics. The remaining authors declare that the research was conducted in the absence of any commercial or financial relationships that could be construed as a potential conflict of interest. Mention of trade name proprietary product or specified equipment in this article is solely for the purpose of providing specific information and does not imply recommendation or endorsement by the authors or their respective institutions.

## Footnotes

1 http://www.ncbi.nlm.nih.gov/tools/primer-blast

## Supplementary Material

FILE S1 Sequencing depth and accession numbers of cattle whole genome sequences.

FIGURE S1 Distribution of visual scores of navel length at yearling in 745,466 Nellore steers.
