# Supplementary figures and images for: Association of Copy Number Variation at Intron 3 of HMGA2 With Navel Length in Bos indicus
Source: Front Genet. 2018 Dec 7;9:627. doi: 10.3389/fgene.2018.00627 (PMC6292862; doi:10.3389/fgene.2018.00627)

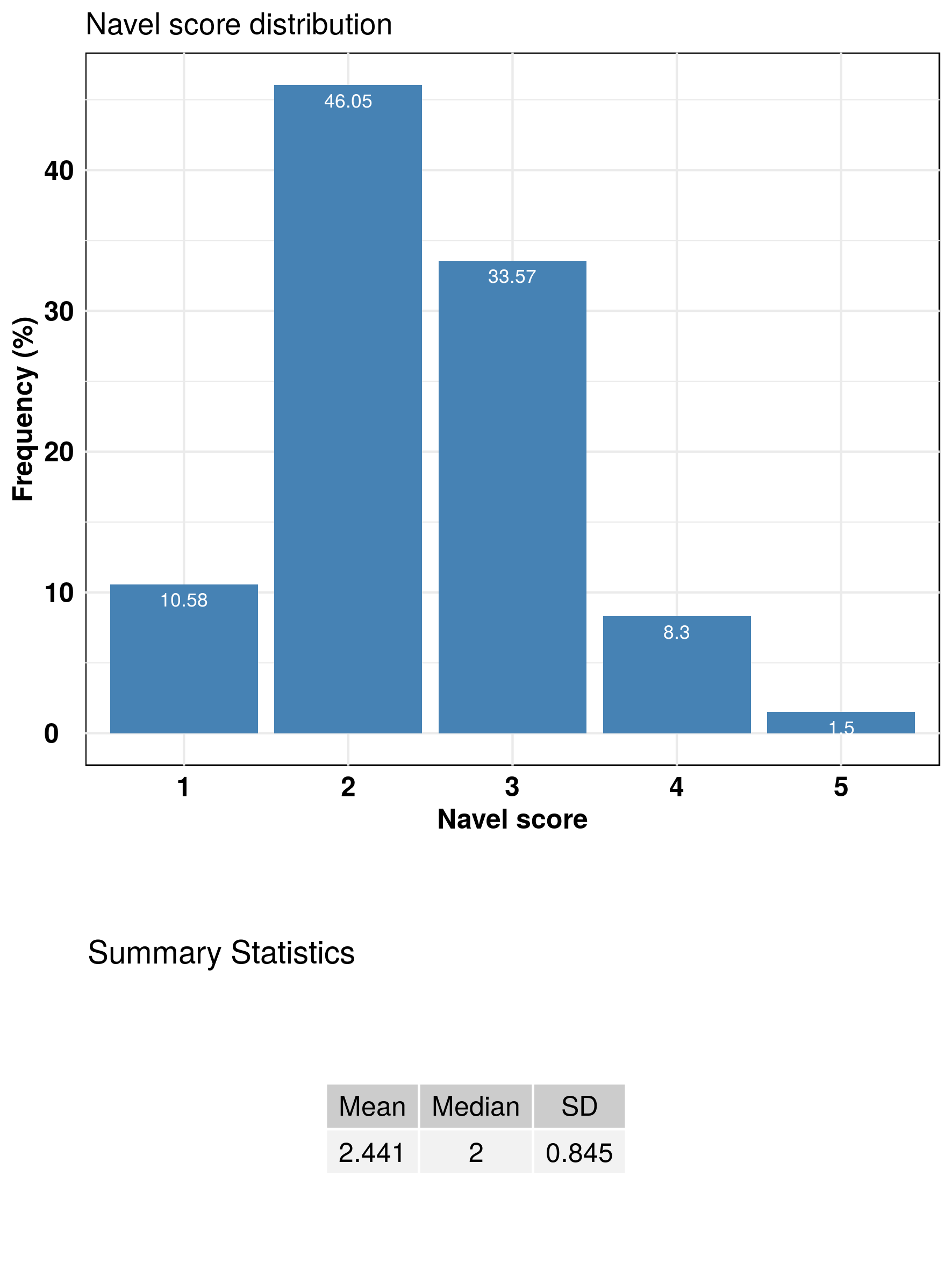

Supplement: FIGURE S1 — Distribution of visual scores of navel length at yearling in 745,466 Nellore steers. [file Image_1.TIF]
